# A Dual-Population-Based NSGA-III for Constrained Many-Objective Optimization

**DOI:** 10.3390/e25010013

**Published:** 2022-12-21

**Authors:** Huantong Geng, Zhengli Zhou, Junye Shen, Feifei Song

**Affiliations:** 1 School of Computer Science, Nanjing University of Information Science and Technology, Nanjing 210044, China; 2 School of Information Technology, Jiangsu Open University, Nanjing 210036, China

**Keywords:** constrained many-objective optimization, evolutionary algorithm, dual-population, coevolution, ε-constraint handling

## Abstract

The main challenge for constrained many-objective optimization problems (CMaOPs) is how to achieve a balance between feasible and infeasible solutions. Most of the existing constrained many-objective evolutionary algorithms (CMaOEAs) are feasibility-driven, neglecting the maintenance of population convergence and diversity when dealing with conflicting objectives and constraints. This might lead to the population being stuck at some locally optimal or locally feasible regions. To alleviate the above challenges, we proposed a dual-population-based NSGA-III, named DP-NSGA-III, where the two populations exchange information through the offspring. The main population based on the NSGA-III solves CMaOPs and the auxiliary populations with different environment selection ignore the constraints. In addition, we designed an ε-constraint handling method in combination with NSGA-III, aiming to exploit the excellent infeasible solutions in the main population. The proposed DP-NSGA-III is compared with four state-of-the-art CMaOEAs on a series of benchmark problems. The experimental results show that the proposed evolutionary algorithm is highly competitive in solving CMaOPs.

## 1. Introduction

Constrained multi-objective optimization problems (CMOPs) widely exist in the real world, such as the design of the water distribution network [1] and the vehicle routing problem with time windows [2]. In this paper, a dual-population-based NSGA-III with an auxiliary population that ignores constraints is proposed. The offspring of its auxiliary population can guide the evolution of the main population.

In the last decade, several new methods have been proposed to solve unconstrained multi/many-objective optimization problems, such as a new competitive mechanism [3], adaptive operator selection [4], and adaptive weight vector generation strategy [5]. In recent years, CMOPs have attracted increasing research interest in the field of evolutionary computation. Constrained multi-objective evolutionary algorithms (CMOEAs), such as PPS [6], ToP [7], and c-DPEA [8], have achieved good performance on CMOPs. However, CMaOPs involve both complex constraints and high-dimensional objective spaces, so it is inefficient to solve CMaOPs directly with existing CMOEAs. Specifically, the difficulties in solving CMaOPs are as follows: first, even in the unconstrained case, balancing convergence and diversity in a high-dimensional objective space is difficult to achieve [9]. Although CMOEAs can handle constraints, they cannot efficiently select excellent solutions in a high-dimensional objective space. Meanwhile, the algorithm should balance the search between feasible and infeasible regions. Particularly, some CMaOPs have complex constraints. It is difficult for individuals to cross multiple disjoint infeasible regions to approach the constraint Pareto front (CPF), which leads to poor convergence and diversity of the population [10]. In addition, most evolutionary algorithms just simply push solutions toward the feasible boundary, and it is difficult to balance the search between feasible and infeasible regions [11]. This may cause the population to fall into locally optimal or locally feasible areas, especially when the feasible region is discrete, narrow, or far from the unconstrained Pareto front (UPF) [12]. To alleviate the above problems, this article proposes a dual-population-based constrained many-objective evolutionary algorithm, named DP-NSGA-III. It includes two collaborative and complementary populations. The main characteristics of DP-NSGA-III are as follows:

1. To take advantage of the infeasible solution with excellent objectives, we introduce an auxiliary population, and its environmental selection [13] is different from the main population, using *p*-norm to select individuals. Two populations cooperate by exchanging offspring. Each population can evolve more efficiently using information from the other population. Specifically, Population1 is the main population, which finds the CPF by the ε-constraint handling method. Population2 with less convergence pressure is the auxiliary population, which will eventually converge to UPF due to the neglected constraints.
2. From the point of view of the problem, neither population solves the original CMaOPs. Since the ε-constraint boundary replaces the original constraint, Population1 solves a problem with looser constraints than the original problem and is equivalent to the original problem at the end of evolution. Population2 solves the unconstrained problem corresponding to the original problem. This strategy indirectly solves the original CMaOPs.
3. To make the main population of DP-NSGA-III infeasibility-driven, we design a monotonically decreasing ε-constraint handling function that takes no additional parameters. That is, the ε-bounded boundary is larger in the early period and close to 0 in the late period. Therefore, this function has a greater impact in the early evolutionary period. The function aims to involve partially infeasible solutions in the evolution, balancing convergence and diversity by using excellent infeasible solutions.

The rest of this article is organized as follows: Section 2 briefly introduces the existing CMOEAs or CMaOEAs. The details of the proposed DP-NSGA-III are described in Section 3. Section 4 and Section 5 present the experimental setup and experimental results, respectively. Finally, the conclusions and future work are given in Section 6.

## 2. Related Work

This section presents some representative CMOEAs and CMaOEAs from the perspective of constraint handling methods. According to the research trend of constraint handling techniques in evolutionary algorithms in earlier years, early constraint handling techniques can be broadly categorized into (1) penalty function-based methods [14]; (2) feasibility-oriented approaches [15]; (3) stochastic ranking [16]; (4) ε-constrained methods [17]; (5) multi-objective optimization-based approaches [18]; and (6) hybrid methods [19].

The constraint-dominance principle(CDP) [15] was one of the earlier well-known constraint handling methods. NSGA-II [15] employs the CDP to compare pairs of solutions. For two infeasible solutions, the one with less constraint violation is better; the two feasible solutions are still compared by the Pareto dominance. Both C-NSGA-III [20] and C-MOEA/D [20] are well-known representative algorithms that embed this technique. Fan et al. [21] proposed an angle-based CDP, which considers the ratio of feasible solutions and the angular relationship between individuals. Among the above algorithms, NSGA-III is designed for many-objective optimization problems, so C-NSGA-III can solve some CMaOPs.

In recent years, some evolutionary algorithms based on multiple stages have been designed to solve CMOPs. PPS [6] divides the search process into two stages: Push and Pull. The Push phase does not consider constraints, which helps to cross large infeasible areas and push the population to UPF. In the Pull phase, an improved ε-constraint handling method is used to pull the population back to the CPF. Liu et al. [7] proposed a phased framework ToP. In ToP, the first stage uses a weighted sum to transform the CMOP into an unconstrained single objective optimization problem to find a promising feasible region. The second stage uses the existing CMOEA to find the CPF of the original problem. CMOEA-MS [22] is also a two-stage evolutionary algorithm, which adjusts the fitness evaluation strategies during the evolutionary process to adaptively balance objective optimization and constraint satisfaction.

In addition, dual-population-based or coevolutionary technique has also been applied to CMOEAs or CMaOEAs. c-DPEA [8] is a typical dual-population-based evolutionary algorithm. Population1 uses an adaptive penalty function named saPF to handle infeasible solutions, and Population2 is oriented to feasibility. At the same time, an adaptive fitness function called bCAD is designed to better balance the convergence and diversity in the evolution process. Although c-DPEA can handle CMOPs well, it is inefficient in solving CMaOPs. C-TAEA [11] is an evolutionary algorithm with two archives. It maintains two collaborative archives simultaneously. The convergence-oriented archive (CA) aims to optimize constraints and objectives to ensure feasibility and convergence, while the diversity-oriented archive (DA) mainly tends to explore the areas that have not been exploited by the CA without considering feasibility. It is worth noting that C-TAEA is one of the few evolutionary algorithms designed for high-dimensional constrained objective spaces.

## 3. Proposed Algorithm: DP-NSGA-III

**Figure 1:**
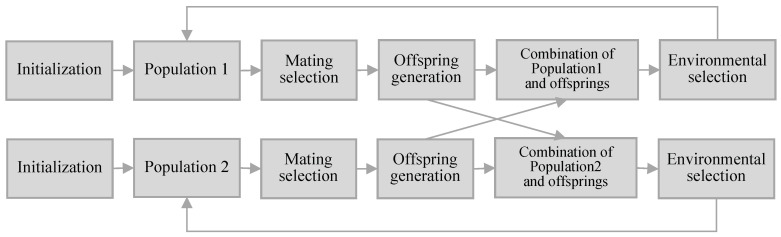
The procedure of DP-NSGA-III.

This section presents the proposed dual-population-based many-objective evolutionary algorithm, named DP-NSGA-III. The procedure of DP-NSGA-III is shown in Figure 1. DP-NAGS-III is a cooperative evolutionary algorithm that evolves two populations, namely, Population1 and Population2. The populations in DP-NSGA-III are evolved separately, only sharing all the offspring in each generation, where Population1 uses the ε-constraint handling method and Population2 ignores the constraint. Population2 can provide solutions for Population1 that violate the constraint but have excellent objective values to participate in evolution. The final output population of the algorithm is Population1. The ε-constraint handling method and the detailed evolution process of each population will be described below.

### 3.1. Constraint Handling

A CMOP can be mathematically defined as:

(1) $$\begin{array}{cc} \min\;F(x) & =\left(f_{1}\left(x\right),f_{2}\left(x\right),\cdots ,f_{M}\left(x\right)\right)\\ s.t.\;g(x) & =\left(g_{1}\left(x\right),\ldots ,g_{k}\left(x\right)\right)\leq 0\\ \mathrm{where}\;x & =\left(x_{1},\ldots ,x_{D}\right)\in \Omega \end{array}$$

where x is a decision vector in decision space Ω. F(x) is the objective vector that consists of *M* conflicting objective functions in objective space. $g_{j}\left(x\right)$ is the *j*-th inequality constraint. A solution x is called a feasible solution in case g(x)≤0; otherwise, it is an infeasible solution. In particular, when M>3, the problem is called a constrained many-objective optimization problem (CMaOP), and the objective space is said to be high-dimensional.

In this article, the constraint violation is defined as the normalized violation of x on all constraints:

(2) $$cv\left(x\right)=\frac{1}{q}\sum_{i=1}^{q}\frac{G_{i}\left(x\right)}{\max\left\{\max_{x\in P}\left\{G_{i}\left(x\right)\right\},1\right\}}$$

where *P* denotes the initial population, and $G_{i}\left(x\right)$ represents the degree of the constraint violation of x on the *i*-th constraint:

(3) $$G_{i}\left(x\right)=\max\left\{g_{j}\left(x\right),0\right\},i=1,2,\cdots ,q$$

To avoid a population excessively weighted towards feasibility and to be able to involve infeasible solutions with excellent objective values in evolution, a parameter-free ε-constraint boundary function to deal with constraints is proposed, with the detailed formulation given as follows:

(4) $$\varepsilon \left(t\right)=\frac{cv_{0}\left(x\right)}{1+e^{20\left(\frac{t}{MaxT}-0.4\right)}},t=1,2,\cdots ,MaxT$$

where $cv_{0}\left(x\right)$ is the constraint violation of population initialization, and *t* is the number of iterations. The constraint boundary is a monotonically decreasing function with an initial value approximating $cv_{0}\left(x\right)$. As the population iterates, the constraint boundary value approaches 0. Therefore, at the end of evolution, this ε-constraint handling method is consistent with Deb’s CDP, i.e., feasible solutions are preferred over infeasible solutions.

Based on the above designed ε(t), the evolutionary algorithm solves the problem as shown below:

(5) $$\begin{array}{cc} \min\;f(x) & =\left(f_{1}\left(x\right),f_{2}\left(x\right),\cdots ,f_{M}\left(x\right)\right)\\ s.t.\;g(x) & =\left(g_{1}\left(x\right),\ldots ,g_{k}\left(x\right)\right)\leq \varepsilon \left(t\right)\\ \mathrm{where}\;\varepsilon (t) & \to 0 \end{array}$$

The problem is equivalent to the original CMaOPs with relaxed constraints in the early stage. In the late stage, since g(x)→0, it is equivalent to the original problem.

Based on the proposed ε-constraint handling method, the proposed evolutionary algorithm will solve Equation (1) indirectly. The main population solves the problem represented by Equation (5), and the auxiliary population solves the unconstrained problem corresponding to Equation (1).

### 3.2. Mating Selection and Offspring Reproduction

Population1 uses the ε-constraint handling method to deal with constraints. To make the offspring satisfy the ε-constraint boundary to the greatest extent, Population1 prefers an ε-feasible solution to an ε-infeasible solution, or a solution with a small constraint violation to a solution with a large constraint violation as the parent and puts it into the mating pool1. Algorithm 1 describes the above tournament selection procedure in detail.

Population2 does not consider constraints, so the nondominated solution is preferred as the parent and puts it into the mating pool2. If the two solutions do not dominate each other, then the selection is made by survival score [13].

Once the mating pool is constructed, the offspring of the population of size *N* is created using simulated binary crossover (SBX) [23] and polynomial mutation (PM) [24] operators. Finally, coevolution is achieved by merging Population1 (or Population2) with offspring 1 and offspring 2.
**Algorithm1:** Tournament selection of Population1
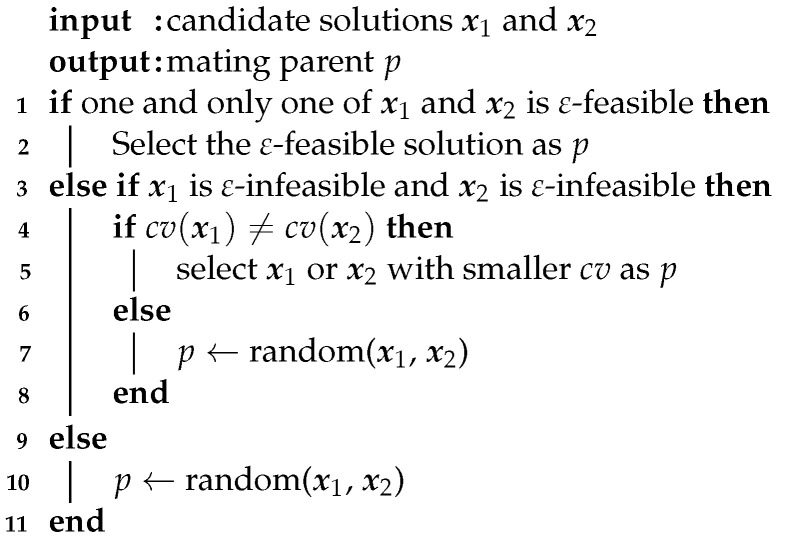


### 3.3. Environmental Selection of Population1

The environment selection of Population1 is based on NSGA-III combined with the ε-constraint handling method. This stage will select *N* outstanding individuals from Population1, offspring1, and offspring2 as new Population1.

The simplest case is that the number of ε-feasible solutions $N_{f}$ is less than *N*. Then, the new population consists of all ε-feasible solutions and $N-N_{f}$ solutions with the smallest constraint violation.

Otherwise, $N_{f}$ is more than *N*. The reference-point-based nondominated sorting with two steps is performed. First, different nondominated levels are classified by using the nondominated sorting based on M+1 objectives: $\left(f_{1}\left(x\right),f_{2}\left(x\right),\cdots ,f_{M}\left(x\right),cv\left(x\right)\right)$. Note that the constraint violation is treated as a new objective in this process. Then, Population1 is distinguished into $F_{1},F_{2},\cdots ,F_{k},\cdots ,F_{n}$ and satisfies $|F_{1}\cup F_{2}\cup \cdots \cup F_{k-1}|\leq N$, $|F_{1}\cup F_{2}\cup \cdots \cup F_{k}|>N$. Second, reference-point-based elite selection is used to maintain population diversity. $N-|F_{1}\cup F_{2}\cup \cdots \cup F_{k-1}|$ solutions are selected in $F_{k}$ based on the association between solutions and the predefined reference points (see Algorithm 2).

It is worth noting that, since the environmental selection of Population1 tends to ε-feasible solutions, the excellent infeasible solutions in offsping2 also have the possibility of entering the next generation. That is, the ε-constraint handling method provides the possibility of retaining the excellent offspring in coevolution.
**Algorithm2:** Reference-point-based nondominated sorting
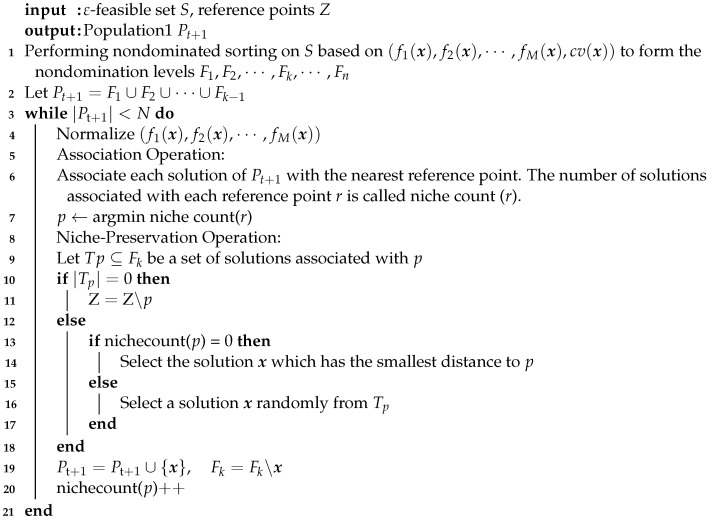


### 3.4. Environmental Selection of Population2

Population2 does not consider the constraint and eventually aims to converge to the UPF. Its environment selection [13] evaluates the solutions by *p*-norm, which can well distinguish the solutions in the high-dimensional objective space. This stage will select *N* outstanding individuals from Population2, offspring1, and offspring2 as new Population2. First, the nondominated levels are still divided by nondominated sorting. The following steps will be completely different from Population1. In each generation, Population2 is assigned fitness according to a fitness function called survival score. The survival score is composed of the ratio of the diversity score and the convergence score.

#### 3.4.1. The *p*-Norm of the First Front $F_{1}$

**Figure 2:**
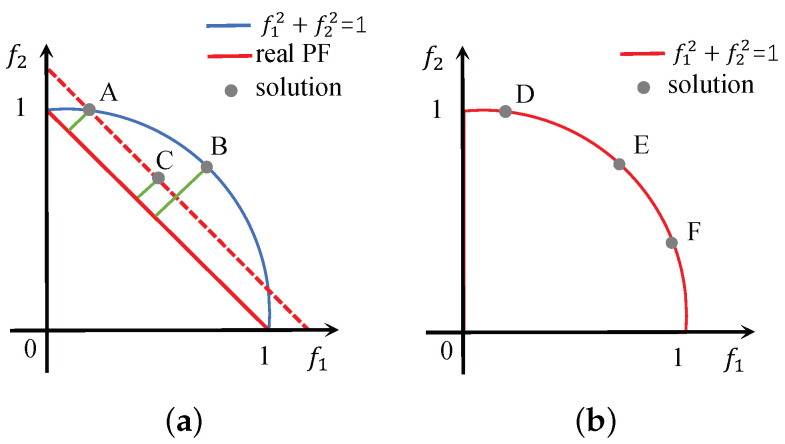
Solutions on different contours. (**a**) The real PF is the contour of 1-norm. (**b**) $F_{1}$ is the contour of 2-norm.

The distance of the solution to the real PF reflects the convergence. However, the real PF is unknown, so the convergence is usually expressed as the Euclidean distance from the solution to the ideal point $Z^{\min}=\left(0,0\right)$, i.e., the 2-norm of the solution. In Figure 2a, B is located at $f_{1}+f_{2}=1$, C is located at $f_{1}+f_{2}=1.2$, and A is located at the intersection of the red dashed line and the blue line. According to the Euclidean distance, since points A and B lie on the same circle, $1={∥A∥}_{2}={∥B∥}_{2}>{∥C∥}_{2}$. However, the green line shows that A and C have the same distance to the real PF (assuming we know it), while B is the farthest. Therefore, in this case, 2-norm does not correctly represent the distance from the solution to the real PF. However, 1-norm can derive the correct distance relationship because A and C lie on 1-norm’s contour $f_{1}^{1}+f_{2}^{1}=1.2$ and B is on the outside of the contour, i.e., $1.2={∥A∥}_{1}={∥C∥}_{1}<{∥B∥}_{1}$.

Therefore, a suitable *p*-norm is chosen to measure the convergence of the solution. Since the real PF is unknown, assume that the shape of the first front $F_{1}$ is the same as real PF and use the *p*-norm of $F_{1}$ to replace the *p*-norm of the real PF. A method to find the *p*-norm of $F_{1}$ is given below.

After normalizing the populations, the solution $x=(x_{1},\cdots ,x_{D})$ in the $F_{1}$ can be regarded as on the unitary hypersurface, satisfying the unique contour equation ${\left(x_{1}^{p}+\cdots +x_{D}^{p}\right)}^{1/p}=1$. This equation is equivalent to finding the zeros of $h\left(p\right)=ln\left(x_{1}^{p}+\cdots +x_{D}^{p}\right)$. The function is second order derivable and can be solved by Halley’s method [25] as follows:

(6) $$p_{n+1}=p_{n}-\frac{h\left(p_{n}\right)}{h^{\prime }\left(p_{n}\right)}{\left(1-\frac{h\left(p_{n}\right)h^{\prime \prime }\left(p_{n}\right)}{2{\left(h^{\prime }\left(p_{n}\right)\right)}^{2}}\right)}^{-1},k=0,1,2,\cdots$$

The *p*-norm of $F_{1}$ can be solved by substituting any solution in $F_{1}$ into Equation (6). Figure 2b was used to verify the correctness of Equation (6), which is the case of p=2. $D\left(\cos\frac{7\pi }{16},\sin\frac{7\pi }{16}\right)$, $E\left(\cos\frac{\pi }{4},\sin\frac{\pi }{4}\right)$ and $F\left(\cos\frac{\pi }{8},\sin\frac{\pi }{8}\right)$ are the normalized solutions in $F_{1}$. Let the initial value $p_{0}=1$, then substitute D, E or F into Equation (6) to obtain $p=p_{2}\approx 2$ in only two iterations. p≈2 is consistent with the actual situation, since the three solutions lie on the contour of the 2-norm.

The computational complexity of Halley’s method is O(MT), where *T* is the number of iterations, generally around 3, much smaller than the size of the population *N*. Therefore, there is no impact on the time complexity of the algorithm.

#### 3.4.2. Survival Score

Once the *p*-norm of the first nondominated front $F_{1}$ is calculated using Equation (6), we can measure both the convergence and diversity of $F_{1}$ [13] accordingly:

(7) $$\begin{array}{cc} \mathrm{convergence}(x) & ={∥f^{n}\left(x\right)-Z^{\min}∥}_{p}={∥f^{n}\left(x\right)∥}_{p} \end{array}$$

(8) $$\begin{array}{cc} \;\mathrm{diversity}\left(x,F_{1}\right) & =\min_{y\neq x\in F_{1}}{∥f^{n}\left(x\right)-f\left(y\right)∥}_{p} \end{array}$$

The convergence score of an individual is the Minkowski distance of the individual to the ideal point $Z^{\min}=\left(0,0\right)$. Since the ideal point is the origin, the convergence is the *p*-norm of the individual. The diversity score of an individual is computed as the minimum Minkowski distance with the other solutions in the front $F_{1}$. The survival score of each solution $x\in F_{1}$ consists of the ratio of diversity to convergence as follows:

(9) $$\mathrm{survival}\mathrm{score}\left(x\right)=\frac{\mathrm{diversity}\left(x,F_{1}\right)}{\mathrm{convergence}(x)}$$

The procedure for calculating the survival score of the solution in $F_{1}$ is shown in Algorithm 3. Note that, if the solution $x\notin F_{1}$, then its survival score is the reciprocal of the convergence score.
**Algorithm3:** Survival Score of $F_{1}$
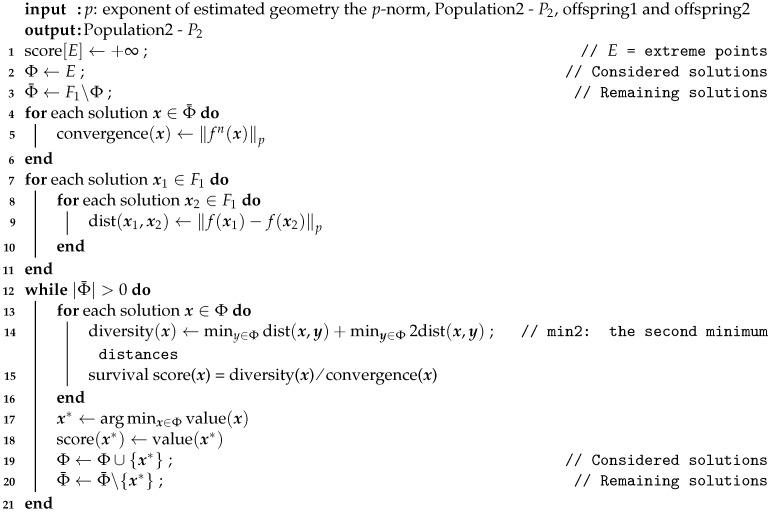


### 3.5. Discussion

**Figure 3:**
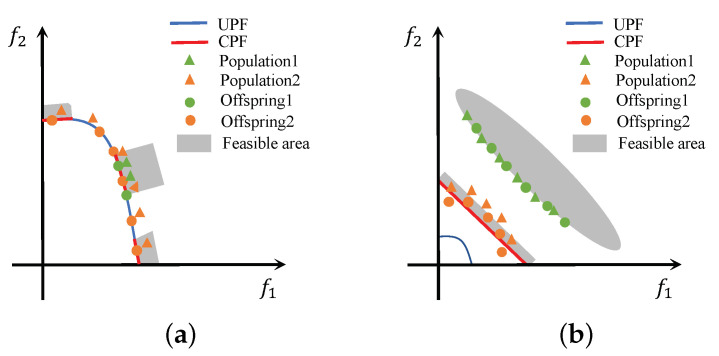
Role of the Population2. (**a**) Offspring2 with good diversity; (**b**) Offspring2 with good convergence.

Population2 has a different population distribution than Population1 because it ignores the constraint. On the one hand, Population2 can provide offspring with good diversity. In Figure 3a, Population1 is trapped in a local optimum, and its Offspring1 have difficulty finding other feasible areas. Population2 is widely distributed around the UPF due to ignoring constraints. Through the coevolution of offspring combinations, the better-diversified Offspring2 can lead Population1 to explore other feasible regions. On the other hand, Population2 can provide offspring with good convergence. In Figure 3b, Population1 falls into a local optimum away from the CPF and Population2, which does not consider constraints and explores towards the UPF. The Offspring2 near the CPF can pull Population1 near the CPF.

### 3.6. Time Complexity of DP-NSGA-III

The proposed ε-constraint handling method has no effect on the time complexity of NSGA-III, so the time complexity of Population1 is still $O(MN^{2})$ [9,26].

The time complexity of Population2 is influenced by nondominated sorting and the calculation of survival score. The time complexity of nondominated sorting is the same as Population1 with $O(MN^{2})$[26]. The computational complexity of calculation of survival score of $F_{1}$(Algorithm 3) is $O\left(MN^{2}\right)+O\left(N^{3}\right)$—specifically, $O(MN^{2})$ for the loop in lines 7–10, and $O(N^{3})$ for the loop in lines 12–21. The survival score of the other nondominated levels is calculated consistently with lines 4–6 as O(MN).

Therefore, the time complexity of DP-NSGA-III is $O\left(MN^{2}\right)+O\left(N^{3}\right)$.

## 4. Experimental Setup

### 4.1. Test Instances

In this article, C-DTLZ [20], DC-DTLZ [11], and MW [27] suites have been selected for examining the performance of DP-NSGA-III. The C-DTLZ suite is based on the DTLZ suite, and four constraints are added to the objective space, while the DC-DTLZ suite introduces three types of constraints in the decision space. The DC-DTLZ suite involves many local optima in infeasible regions, which causes the algorithm may easily fall into some infeasible local optima and cannot enter the feasible region. The objective number of the C-DTLZ suite and the DC-DTLZ suite is scalable. The MW suite was recently proposed, and it contains a variety of features, such as a small feasibility area, multiple complex nonlinear constraints, a scalable number of objectives, and high-dimensional decision vectors. Since some of the test functions in the MW suite have a fixed objective number, in this section, we only test MW4, MW8, and MW14 with variable objective space dimensions.

According to the relationship between UPF and CPF, the above test problems can be divided into three categories [20]:

Type-I: The CPF is the same as the UPF. MW4, MW14, C1DTLZ1, C1DTLZ3, DC2DTLZ1, and DC2DTLZ3 are Type-I problems.

Type-II: Infeasible area makes the UPF partly feasible and the CPF is a part of the UPF. MW8, C2DTLZ2, DC1DTLZ1, and DC1DTLZ3 are Type-II problems.

Type-III: The UPF is completely infeasible and the CPF is located on the boundary of the feasible region. C3DTLZ4, DC3DTLZ1, and DC3DTLZ3 are Type-III problems.

### 4.2. Performance Metrics

To measure the performance of DP-NSGA-III and other peer CMaOEAs, the following two commonly used metrics are adopted. Furthermore, the Wilcoxon rank sum test with a significance level of 0.05 is adopted to perform statistical analysis.

(1) Inverted Generational Distance (IGD) [28]: Given $P^{*}$ as a set of points uniformly sampled along the PF and *P* as the set of solutions obtained from an evolutionary algorithm. The IGD value of *P* is calculated as
  (10) $$\mathrm{IGD}\left(P,P^{*}\right)=\frac{\sum_{x\in P^{*}}\mathrm{dist}\left(x,P\right)}{\left|P^{*}\right|}$$
  where dist(x,P) is the Euclidean distance between x and its nearest neighbor in *P*.

(2) Hypervolume (HV) [29]: Let $z^{r}={\left(z_{1}^{r},\cdots ,z_{m}^{r}\right)}^{T}$ be the worst point dominated by all the Pareto optimal objective vectors. The HV of *P* is defined as the volume of the objective space dominated by solutions in *P* and bounded by $z^{r}$

(11) $$\mathrm{HV}\left(P\right)=\mathrm{VOL}\left(\underset{z\in P}{⋃}\left[z_{1},z_{1}^{r}\right]\times \cdots \times \left[z_{m},z_{m}^{r}\right]\right)$$

### 4.3. Algorithms for Comparison

Four state-of-the-art and representative CMaOEAs were chosen for performance comparison: C-NSGA-III [20] (Abbreviated as NSGA-III), C-TAEA [11], DCNSGA-III [30], and MOEA/D-2WA [31]. NSGA-III and C-TAEA have been briefly introduced in Section 2. Thus, we briefly describe others’ basic ideas as follows:

DCNSGA-III: It is an improved NSGA-III that uses dynamic multi-objective techniques to solve CMaOPs. It is more suitable for processing CMaOPs than NSGA-III due to the upgrade of the constraint processing method.
MOEA/D-2WA: It is a modified MOEA/D using two-type weight adjustments. During the search, the number of infeasible weights is dynamically lowered to help infeasible solutions with greater convergence transcend the infeasible areas, as well as to help infeasible solutions with better diversity identify many feasible sub regions.

### 4.4. Parameter Settings

(1) Algorithms and reproduction operators: To make a fair comparison and ensure the performance of each algorithm, the parameters of all the compared algorithm and their reproduction operator parameters, such as the simulated binary crossover and polynomial mutation, are set as suggested in their original papers. All the compared algorithms were implemented in PlatEMO [32].
(2) Population size and the number of function evaluations: Table 1 shows the number of reference points and population size of each algorithm for different numbers of objectives *M* [11,27]. The number of function evaluations is the termination condition of all algorithms [11,27]. The details are shown in Table 2.

## 5. Experimental Results

This section focuses on analyzing the performance of the proposed DP-NSGA-III with the peer CMaOEAs on three test suites. Note that the following population distribution plots or Parallel coordinate plots (PCPs) are taken for the median IGD of 30 independent experiments.

### 5.1. Performance on C-DTLZ Suite

Wilcoxon’s test results on C-DTLZ suite are summarized in Table 3. According to the IGD values, the results show that DP-NSGA-III is significantly better than its competitors on at least 7/16 and at most 14/16 test problems. The results indicate that DP-NSGA-III performs much better than its rivals on at least 8/16 and at most 12/16 test problems, based on the HV values.

Table A1 and Table A2 show the mean value and standard deviation of the IGD and HV values obtained from the above-mentioned five algorithms over 30 runs on the C-DTLZ suite with 3, 5, 10, and 15 objectives. The C-DTLZ suite is moderately difficult, and the proposed algorithm achieves good results on all but C3DTLZ4.

**Figure 4:**
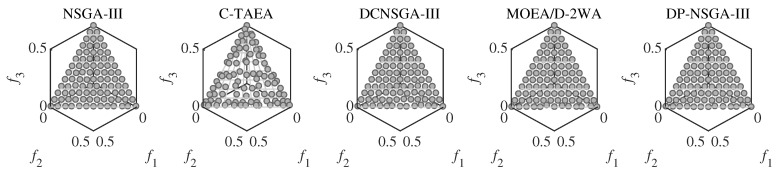
Scatter plots of the population obtained by the peer algorithms and DP-NSGA-III on 3-objective C1DTLZ1 (median IGD value). The grey area is the real Pareto front.

For C1DTLZ1, only a narrow section near PF is feasible. Both the DP-NSGA-III and comparison algorithms converge on this problem. Figure 4 shows that differences in performance metric are mainly influenced by the uniformity of population distribution. C-TAEA has a messy population distribution and therefore has the worst performance metric.

**Figure 5:**
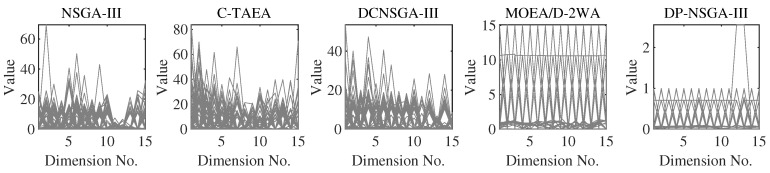
PCPs of the populations obtained by the peer algorithms and DP-NSGA-III on 15-objective C1DTLZ3 (median IGD value).

The C1DTLZ3 problem has a highly multimodal landscape and infeasible region barriers that pose a challenge to the convergence of CMaOEA. Populations have difficulty crossing infeasible barriers and are easily trapped in localized feasible areas. The IGD of the proposed DP-NSGA-III is better than other peer algorithms by more than an order of magnitude in all four dimensions. The global optimal value of C1DTLZ3 is between 0 and 1. Figure 5 indicates that DP-NSGA-III converges to the global optimum except for a very small number of solutions. The other algorithms only converge to the boundary of the local feasible region and fall into the local optimum with a large number of solutions with objective values greater than 1. For DP-NAGA-III, the auxiliary populations that ignore the constraints help the main population to cross a huge infeasibility region.

The CPF of C2DTLZ2 is part of the UPF, and the areas are not connected. This problem mainly tests the diversity maintenance ability of the algorithm. Most evolutionary algorithms found the CPF, and DP-NSGA-III performed relatively well.

**Figure 6:**
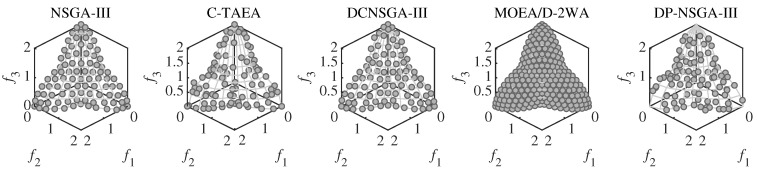
Scatter plots of the population obtained by the peer algorithms and DP-NSGA-III on 3-objective C3DTLZ4 (median IGD value).The grey area is the real Pareto front.

The CPF of C3DLTZ4 is far from the UPF and is the boundary of the feasible area. In this issue, DP-NSGA-III was able to find CPF, but the population diversity was poor. Figure 6 shows the most uniform population distribution obtained by MOEA/D-2WA with two-type weight.

### 5.2. Performance on DC-DTLZ Suite

Wilcoxon’s test results on DC-DTLZ suite are summarized in Table 4. According to the IGD values, the results show that DP-NSGA-III is significantly better than its competitors on at least 14/22 and at most 20/22 test problems. The results indicate that DP-NSGA-III performs much better than its rivals on at least 12/22 and at most 21/22 test problems, based on the HV values.

Table A3 and Table A4 show the mean value and standard deviation of the IGD and HV values obtained from the above-mentioned five algorithms over 30 runs on the DC-DTLZ suite with 3, 5, 10, and 15 objectives.

**Figure 7:**
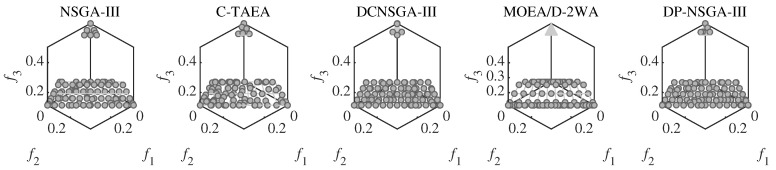
Scatter plots of the population obtained by the peer algorithms and DP-NSGA-III on 3-objective DC1DTLZ1 (median IGD value).The grey area is the real Pareto front.

DC1DTLZ1 and DC1DTLZ3 are the Type-II problems. The CPF consists of multiple disjoint feasible areas. The obtained population is easily trapped in the local optimal region, and it is difficult to find the complete PF. Performance metrics show that DP-NSGA-III has a greater advantage over DC1DTLZ3 than DC1DTLZ1. Figure 7 illustrates that the population of DP-NSGA-III is evenly distributed on two separated PFs of DC1DTLZ1, while MOEA/D-2WA fails to find the PF above.

**Figure 8:**
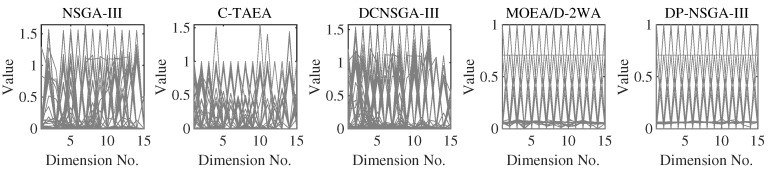
PCPs of the populations obtained by the peer algorithms and DP-NSGA-III on 15-objective DC2DTLZ3 (median IGD value).

DC2DTLZ1 and DC2DTLZ3 belong to the Type-I problems. Although it has the same CPF as UPF, the infeasibility region is huge and only the region near PF is feasible. Moreover, there exist many local optima in infeasible regions, making it much harder for solutions to escape. DP-NSGA-III achieved the best results in all four dimensions of DC2DTLZ1. Figure 8 demonstrates that DP-NSGA-III and MOEA/D-2WA perform best on DC2DTLZ3; in particular, the convergence and diversity of DP-NSGA-III are improved over NSGA-III and DCNSGA-III. NSGA-III and DCNSGA-III have objective values greater than 1 in multiple dimensions, indicating that there are solutions that fail to converge to the global optimum. Ignoring the few solutions with objective values greater than 1, the polyline in parallel coordinates for C-TAEA was not uniformly distributed compared to DP-NSGA-III, indicating an uneven distribution of the population.

**Figure 9:**
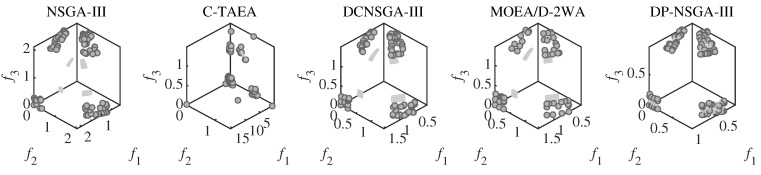
Scatter plots of the population obtained by the peer algorithms and DP-NSGA-III on 3-objective DC3DTLZ3 (median IGD value).The grey area is the real Pareto front.

DC3DTLZ1 and DC3DTLZ3 can be generated as Type-III problems, which are a mixture of the first two kinds of test instances. As the number of objectives increases, the feasible region becomes smaller and smaller. Note that no feasible solutions are found for all algorithms on the DC3DTLZ1 problems when M≥10. Figure 9 shows that, except for DP-NSGA-III, all the algorithms fail to jump out of the infeasible region on DC3DTLZ3. DP-NSGA-III has the best convergence and distribution.

### 5.3. Performance on MW Suite

Wilcoxon’s test results on MW suite are summarized in Table 5. According to the IGD values, the results show that DP-NSGA-III is significantly better than its competitors on at least 6/12 and at most 10/12 test problems. The results indicate that DP-NSGA-III performs much better than its rivals on at least 6/12 and at most 10/12 test problems, based on the HV values.

Table A5 and Table A6 show the mean value and standard deviation of the IGD and HV values obtained from the above-mentioned five algorithms over 30 runs on the MW suite with 3, 5, 10, and 15 objectives.

**Figure 10:**
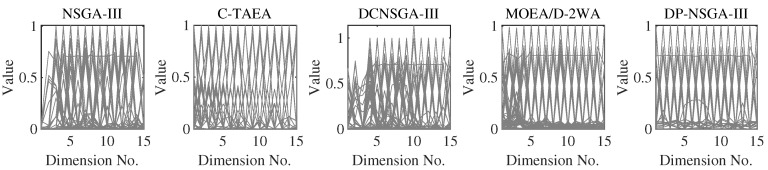
PCPs of the populations obtained by the peer algorithms and DP-NSGA-III on 15-objective MW8 (median IGD value).

**Figure 11:**
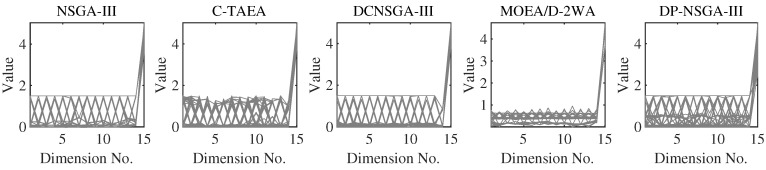
PCPs of the populations obtained by the peer algorithms and DP-NSGA-III on 15-objective MW14 (median IGD value).

MW4, MW8, and MW14 are the problems with the adjustable number of objectives in this suite. From Figure 10, we can see that DP-NSGA-III and MOEA/D-2WA perform comparably and better than other algorithms on the MW8 problem. NSGA-III and DCNSGA-III do not cover areas with a value of 1 in the first few dimensions. Different objectives of the MW14 have different ranges of value domains, with a maximum value of 5 when M=15. Figure 11 shows that MOEA/D-2WA, which relies entirely on the reference vector, has degraded performance in this problem. When M≤14, the population obtained by MOEA/D-2WA fails to find areas with an objective value of 1, while DP-NSGA-III still performs well in higher dimensions.

### 5.4. Stability of DP-NSGA-III

**Figure 12:**
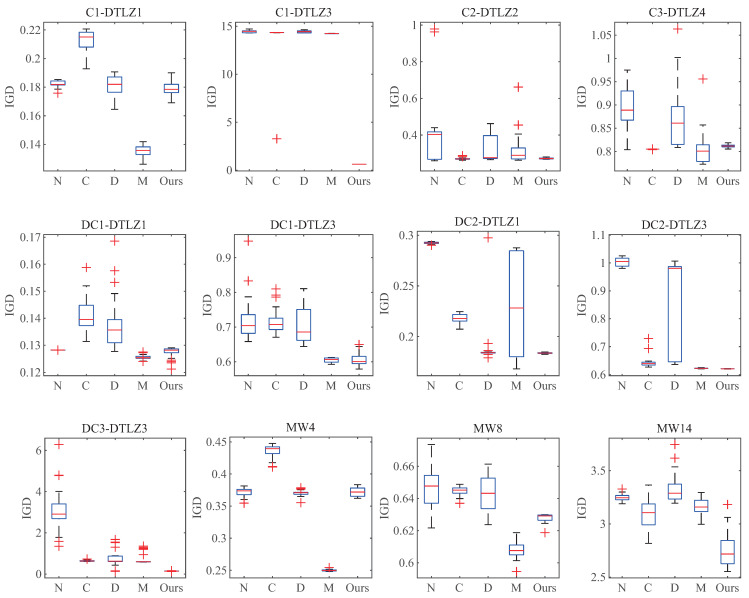
Box plots of IGD for DP-NAGA-III and other peer algorithms on the 15-objective problems.

To evaluate the stability of each algorithm, box plots are used to represent the statistical results of IGD metrics for 30 experiments of the algorithms on each problem (M=15). For convenience, each algorithm is abbreviated with its initials. Each subplot corresponds to the case of M=15 in Table A1, Table A3, and Table A5. Figure 12 shows that, while the other algorithms have more outliers and larger deviations, DP-NSGA-III has only a few outliers and small deviations on individual problems. DP-NSGA-III has the smallest inter-quartile range and the best stability for most problems. In general, DP-NSGA-III has obvious advantages in median, inter-quartile range and outliers, reflecting that DP-NSGA-III can better balance convergence and diversity on 15-dimensional CMaOPs and has better algorithm stability.

## 6. Conclusions

This paper proposed a dual-population-based constrained many-objective evolutionary algorithm, named DP-NSGA-III. Compared with existing CMaOEAs, DP-NSGA-III coevolves by exchanging the offspring of two populations. Two populations use different environmental selection to select superior individuals. Among them, the Auxiliary population deals with CMaOP that ignores constraints, and the main population focuses on solving the original CMaOP. The main population can make full use of the information of infeasible solutions in the auxiliary population to solve the constraint problem more effectively. From the problem perspective, the designed dual population solves the original CMaOPs indirectly. The main population solves the problem of loose constraints and the auxiliary population solves the problem of ignoring constraints. In addition, to improve the ability of the main population to handle constraints, this paper designed a parameter-free ε-constraint handling method. In the main populations driven by infeasible solutions, ε-feasible solutions can provide the impetus for evolution.

The performance of DP-NSGA-III has been studied for a series of suites with complex constraints with up to 15 objectives. Thanks to the proposed constraint handling methods that reduce the difficulty of handling constraints, the experimental results fully demonstrate the competitiveness of DP-NSGA-III in CMaOPs compared to the four state-of-the-art CMaOEAs, especially in DC-DTLZ suite with highly complex constraints. However, if the CPF is not a regular surface, or if the UPF is separated from the CPF, DP-NSGA-III performs poorly. On C3DTLZ4 with both features, DP-NSGA-III converged to CPF, but the populations were not as well distributed as NSGA-III. The solutions provided by the auxiliary population have a side effect instead. In the future, it is necessary to further explore the underlying mechanisms of dual-population and design better dual-population strategies. In terms of environment selection, more efficient scalar methods need to be designed to replace Pareto dominance relationships that make it difficult to distinguish individuals in a high-dimensional objective space.

## Figures and Tables

**Table 1:** The number of reference points and corresponding population size for different numbers of objectives.

Objectives/Dimension No. *M*	Reference No.	Popsize *N*
3	91	92
5	210	212
10	275	276
15	135	136

**Table 2:** The number of maximum function evaluations for different problems.

Problem	M=3	M=5	M=10	M=15
C1DTLZ1	46,000	127,200	276,000	204,000
C1DTLZ3	92,000	318,000	966,000	680,000
C2DTLZ2	23,000	74,200	207,000	136,000
C3DTLZ4	69,000	265,000	828,000	544,000
DC1DTLZ1	69,000	265,000	828,000	544,000
DC1DTLZ3	69,000	265,000	828,000	544,000
DC2DTLZ1	138,000	530,000	1656,000	1088,000
DC2DTLZ3	138,000	530,000	1656,000	1088,000
DC3DTLZ1	69,000	265,000	828,000	544,000
DC3DTLZ3	69,000	265,000	828,000	544,000
MW4	55,200	127,200	165,600	81,600
MW8	55,200	127,200	165,600	81,600
MW14	55,200	127,200	165,600	81,600

**Table 3:** Wilcoxon’s test results on C-DTLZ suite ^1^.

	IGD (−/+/=)	HV (−/+/=)
DP-NSGA-III vs. NSGA-III	2/13/1	4/11/1
DP-NSGA-III vs. C-TAEA	2/13/1	3/11/2
DP-NSGA-III vs. DCNSGA-III	0/14/2	2/12/2
DP-NSGA-III vs. MOEA/D-2WA	6/7/3	6/8/2

^1^ “+”, “−”, or “=” denote the number of the performance of DP-NSGA-III is significantly better than, worse than, or comparable to its peer, respectively.

**Table 4:** Wilcoxon’s test results on DC-DTLZ suite ^1^.

	IGD (−/+/=)	HV (−/+/=)
DP-NSGA-III vs. NSGA-III	1/20/1	2/18/2
DP-NSGA-III vs. C-TAEA	0/19/3	1/21/0
DP-NSGA-III vs. DCNSGA-III	0/19/3	0/17/5
DP-NSGA-III vs. MOEA/D-2WA	4/14/4	2/12/8

^1^ “+”, “−”, or “=” denote the number of the performance of DP-NSGA-III is significantly better than, worse than, or comparable to its peer, respectively.

**Table 5:** Wilcoxon’s test results on MW suite ^1^.

	IGD (−/+/=)	HV (−/+/=)
DP-NSGA-III vs. NSGA-III	3/8/1	2/6/4
DP-NSGA-III vs. C-TAEA	2/10/0	2/10/0
DP-NSGA-III vs. DCNSGA-III	1/9/2	2/7/3
DP-NSGA-III vs. MOEA/D-2WA	6/6/0	1/7/4

^1^ “+”, “−”, or “=” denote the number of the performance of DP-NSGA-III is significantly better than, worse than, or comparable to its peer, respectively.

## Data Availability

Not applicable.

## Abbreviations

The following abbreviations are used in this manuscript:

CMOP	Constrained multi-objective optimization problem
CMaOP	Constrained many-objective optimization problem
CMOEA	Constrained multi-objective evolutionary algorithm
CMaOEA	Constrained many-objective evolutionary algorithm
CDP	Constraint-dominance principle
PF	Pareto front
UPF	Unconstrained Pareto front
CPF	Constrained Pareto front
IGD	Inverted generational distance
HV	Hypervolume
PCP	Parallel coordinate plot

## Appendix A

**Table A1:** IGD values obtained by DP-NSGA-III and other peer algorithms in the C-DTLZ suite ^1^.

Problem	*M*	NSGA-III	C-TAEA	DCNSGA-III	MOEA/D-2WA	DP-NSGA-III
C1DTLZ1	3	2.0316 × 10${}^{-2}$(1.66 × 10${}^{-4}$)=	2.3218 × 10${}^{-2}$(5.64 × 10${}^{-4}$)-	**2.0292 × 10${}^{-2}$(1.01 × 10${}^{-4}$)=**	2.0347 × 10${}^{-2}$(1.06 × 10${}^{-4}$)=	2.0342 × 10${}^{-2}$(9.94 × 10${}^{-5}$)
	5	5.2160 × 10${}^{-2}$(2.95 × 10${}^{-4}$)-	5.9101 × 10${}^{-2}$(8.92 × 10${}^{-4}$)-	5.2108 × 10${}^{-2}$(3.15 × 10${}^{-4}$)-	5.1891 × 10${}^{-2}$(2.70 × 10${}^{-4}$)=	**5.1815 × 10${}^{-2}$(2.52 × 10${}^{-4}$)**
	10	1.0934 × 10${}^{-1}$(3.38 × 10${}^{-3}$)-	1.3960 × 10${}^{-1}$(2.37 × 10${}^{-3}$)-	1.1167 × 10${}^{-1}$(7.28 × 10${}^{-3}$)-	**1.0487 × 10${}^{-1}$(5.67 × 10${}^{-4}$)+**	1.0799 × 10${}^{-1}$(5.86 × 10${}^{-4}$)
	15	1.8281 × 10${}^{-1}$(3.39 × 10${}^{-3}$)-	2.1208 × 10${}^{-1}$(7.95 × 10${}^{-3}$)-	1.8062 × 10${}^{-1}$(7.67 × 10${}^{-3}$)=	**1.3518 × 10${}^{-1}$(4.24 × 10${}^{-3}$)+**	1.7936 × 10${}^{-1}$(4.43 × 10${}^{-3}$)
C1DTLZ3	3	3.2850 × 10${}^{0}$(3.93 × 10${}^{0}$)-	7.3722 × 10${}^{-2}$(1.25 × 10${}^{-2}$)-	5.1328 × 10${}^{0}$(3.85 × 10${}^{0}$)-	4.3406 × 10${}^{0}$(4.00 × 10${}^{0}$)-	**5.4508 × 10${}^{-2}$(2.67 × 10${}^{-5}$)**
	5	4.9508 × 10${}^{0}$(5.52 × 10${}^{0}$)-	8.9646 × 10${}^{0}$(4.88 × 10${}^{0}$)-	5.7600 × 10${}^{0}$(5.56 × 10${}^{0}$)-	6.2536 × 10${}^{0}$(5.77 × 10${}^{0}$)-	**1.6513 × 10${}^{-1}$(5.32 × 10${}^{-5}$)**
	10	5.0854 × 10${}^{0}$(6.58 × 10${}^{0}$)-	1.1983 × 10${}^{1}$(5.15 × 10${}^{0}$)-	6.6092 × 10${}^{0}$(6.80 × 10${}^{0}$)-	1.1406 × 10${}^{1}$(5.59 × 10${}^{0}$)-	**4.2023 × 10${}^{-1}$(4.00 × 10${}^{-4}$)**
	15	1.2705 × 10${}^{1}$(4.54 × 10${}^{0}$)-	1.1425 × 10${}^{1}$(5.39 × 10${}^{0}$)-	1.2603 × 10${}^{1}$(4.75 × 10${}^{0}$)-	1.3781 × 10${}^{1}$(2.49 × 10${}^{0}$)-	**6.2255 × 10${}^{-1}$(9.07 × 10${}^{-4}$)**
C2DTLZ2	3	4.8008 × 10${}^{-2}$(4.54 × 10${}^{-4}$)-	5.7025 × 10${}^{-2}$(1.50 × 10${}^{-3}$)-	4.7951 × 10${}^{-2}$(4.62 × 10${}^{-4}$)-	4.9627 × 10${}^{-2}$(4.03 × 10${}^{-4}$)-	**4.7472 × 10${}^{-2}$(4.66 × 10${}^{-4}$)**
	5	1.3891 × 10${}^{-1}$(2.42 × 10${}^{-4}$)-	1.4714 × 10${}^{-1}$(1.25 × 10${}^{-3}$)-	1.3881 × 10${}^{-1}$(3.48 × 10${}^{-4}$)-	1.3911 × 10${}^{-1}$(6.53 × 10${}^{-4}$)-	**1.3759 × 10${}^{-1}$(6.92 × 10${}^{-4}$)**
	10	3.3032 × 10${}^{-1}$(1.65 × 10${}^{-1}$)-	3.4636 × 10${}^{-1}$(3.60 × 10${}^{-2}$)-	2.6499 × 10${}^{-1}$(2.11 × 10${}^{-3}$)-	2.8945 × 10${}^{-1}$(6.53 × 10${}^{-2}$)=	**2.5890 × 10${}^{-1}$(2.36 × 10${}^{-3}$)**
	15	4.0334 × 10${}^{-1}$(1.69 × 10${}^{-1}$)-	**2.7026 × 10${}^{-1}$(5.04 × 10${}^{-3}$)+**	3.3055 × 10${}^{-1}$(7.04 × 10${}^{-2}$)-	3.3590 × 10${}^{-1}$(1.19 × 10${}^{-1}$)-	2.7237 × 10${}^{-1}$(2.85 × 10${}^{-3}$)
C3DTLZ4	3	2.1730 × 10${}^{-1}$(2.85 × 10${}^{-1}$)-	1.1259 × 10${}^{-1}$(2.48 × 10${}^{-3}$)=	1.4665 × 10${}^{-1}$(1.89 × 10${}^{-1}$)-	**5.7976 × 10${}^{-2}$(9.37 × 10${}^{-5}$)+**	1.4219 × 10${}^{-1}$(4.48 × 10${}^{-2}$)
	5	2.4579 × 10${}^{-1}$(8.96 × 10${}^{-4}$)+	2.9056 × 10${}^{-1}$(2.02 × 10${}^{-3}$)-	2.8371 × 10${}^{-1}$(1.09 × 10${}^{-1}$)-	**2.4406 × 10${}^{-1}$(4.52 × 10${}^{-4}$)+**	2.5222 × 10${}^{-1}$(4.96 × 10${}^{-3}$)
	10	5.7797 × 10${}^{-1}$(4.66 × 10${}^{-2}$)+	6.2424 × 10${}^{-1}$(4.86 × 10${}^{-3}$)-	7.0682 × 10${}^{-1}$(6.66 × 10${}^{-2}$)-	**5.6050 × 10${}^{-1}$(1.92 × 10${}^{-3}$)+**	6.0233 × 10${}^{-1}$(4.34 × 10${}^{-2}$)
	15	8.8829 × 10${}^{-1}$(5.01 × 10${}^{-2}$)-	**8.0495 × 10${}^{-1}$(3.51 × 10${}^{-4}$)+**	8.7126 × 10${}^{-1}$(6.00 × 10${}^{-2}$)-	8.0516 × 10${}^{-1}$(3.61 × 10${}^{-2}$)+	8.2850 × 10${}^{-1}$(6.02 × 10${}^{-2}$)
+/−/=		2/13/1	2/13/1	0/14/2	6/7/3	

^1^ “+”, “−”, or “=” indicates that the corresponding algorithm is significantly better than, worse than, or comparable to DP-NSGA-III. The best average value among these algorithms on each test problem is highlighted in bold.

**Table A2:** HV values obtained by DP-NSGA-III and other peer algorithms in the C-DTLZ suite ^1^.

Problem	*M*	NSGA-III	C-TAEA	DCNSGA-III	MOEA/D-2WA	DP-NSGA-III
C1DTLZ1	3	8.3136 × 10${}^{-1}$(8.55 × 10${}^{-3}$)-	8.3071 × 10${}^{-1}$(6.34 × 10${}^{-3}$)-	8.3558 × 10${}^{-1}$(3.94 × 10${}^{-3}$)=	8.3578 × 10${}^{-1}$(4.04 × 10${}^{-3}$)=	**8.3647 × 10${}^{-1}$(4.30 × 10${}^{-3}$)**
	5	9.7520 × 10${}^{-1}$(3.95 × 10${}^{-3}$)=	9.7465 × 10${}^{-1}$(4.62 × 10${}^{-3}$)=	**9.7625 × 10${}^{-1}$(4.68 × 10${}^{-3}$)=**	9.7527 × 10${}^{-1}$(3.61 × 10${}^{-3}$)=	9.7538 × 10${}^{-1}$(3.26 × 10${}^{-3}$)
	10	9.9394 × 10${}^{-1}$(6.75 × 10${}^{-3}$)-	9.9842 × 10${}^{-1}$(1.32 × 10${}^{-3}$)=	9.9659 × 10${}^{-1}$(3.92 × 10${}^{-3}$)-	9.8515 × 10${}^{-1}$(8.06 × 10${}^{-3}$)-	**9.9867 × 10${}^{-1}$(8.77 × 10${}^{-4}$)**
	15	9.9446 × 10${}^{-1}$(6.76 × 10${}^{-3}$)-	9.9484 × 10${}^{-1}$(7.10 × 10${}^{-3}$)-	9.9862 × 10${}^{-1}$(2.56 × 10${}^{-3}$)-	8.9223 × 10${}^{-1}$(2.59 × 10${}^{-2}$)-	**9.9985 × 10${}^{-1}$(7.57 × 10${}^{-5}$)**
C1DTLZ3	3	3.0072 × 10${}^{-1}$(2.78 × 10${}^{-1}$)-	5.3960 × 10${}^{-1}$(9.57 × 10${}^{-3}$)-	1.8095 × 10${}^{-1}$(2.62 × 10${}^{-1}$)-	2.2632 × 10${}^{-1}$(2.76 × 10${}^{-1}$)-	**5.5855 × 10${}^{-1}$(6.05 × 10${}^{-4}$)**
	5	3.6162 × 10${}^{-1}$(3.96 × 10${}^{-1}$)-	1.7036 × 10${}^{-1}$(3.14 × 10${}^{-1}$)-	2.7564 × 10${}^{-1}$(3.78 × 10${}^{-1}$)-	3.4886 × 10${}^{-1}$(3.91 × 10${}^{-1}$)-	**8.1262 × 10${}^{-1}$(4.19 × 10${}^{-4}$)**
	10	5.8279 × 10${}^{-1}$(4.74 × 10${}^{-1}$)-	8.4921 × 10${}^{-2}$(2.46 × 10${}^{-1}$)-	4.0252 × 10${}^{-1}$(4.69 × 10${}^{-1}$)-	1.9396 × 10${}^{-1}$(3.95 × 10${}^{-1}$)-	**9.6961 × 10${}^{-1}$(2.85 × 10${}^{-4}$)**
	15	9.5280 × 10${}^{-2}$(2.91 × 10${}^{-1}$)-	5.5117 × 10${}^{-2}$(2.10 × 10${}^{-1}$)-	1.1506 × 10${}^{-1}$(3.01 × 10${}^{-1}$)-	3.3022 × 10${}^{-2}$(1.81 × 10${}^{-1}$)-	**9.9059 × 10${}^{-1}$(1.37 × 10${}^{-4}$)**
C2DTLZ2	3	5.0609 × 10${}^{-1}$(1.76 × 10${}^{-3}$)+	5.0209 × 10${}^{-1}$(3.47 × 10${}^{-3}$)-	5.0232 × 10${}^{-1}$(2.22 × 10${}^{-3}$)-	**5.1261 × 10${}^{-1}$(6.12 × 10${}^{-4}$)+**	5.0530 × 10${}^{-1}$(1.20 × 10${}^{-3}$)
	5	**75624 × 10${}^{-1}$(5.87 × 10${}^{-4}$)+**	7.3985 × 10${}^{-1}$(1.77 × 10${}^{-3}$)-	7.5597 × 10${}^{-1}$(6.33 × 10${}^{-4}$)+	7.4867 × 10${}^{-1}$(1.36 × 10${}^{-3}$)+	7.4777 × 10${}^{-1}$(1.18 × 10${}^{-3}$)
	10	8.4100 × 10${}^{-1}$(1.74 × 10${}^{-1}$)-	8.7347 × 10${}^{-1}$(4.96 × 10${}^{-3}$)-	8.9450 × 10${}^{-1}$(1.44 × 10${}^{-3}$)-	8.1762 × 10${}^{-1}$(1.29 × 10${}^{-1}$)-	**8.9540 × 10${}^{-1}$(1.64 × 10${}^{-3}$)**
	15	8.7433 × 10${}^{-1}$(1.84 × 10${}^{-1}$)-	9.3273 × 10${}^{-1}$(3.23 × 10${}^{-3}$)-	9.2978 × 10${}^{-1}$(2.75 × 10${}^{-2}$)-	8.3762 × 10${}^{-1}$(2.39 × 10${}^{-1}$)-	**9.5717 × 10${}^{-1}$(3.37 × 10${}^{-4}$)**
C3DTLZ4	3	7.5074 × 10${}^{-1}$(1.00 × 10${}^{-1}$)-	7.8462 × 10${}^{-1}$(1.02 × 10${}^{-3}$)+	7.7286 × 10${}^{-1}$(6.60 × 10${}^{-2}$)+	**8.0612 × 10${}^{-1}$(8.42 × 10${}^{-5}$)+**	7.6916 × 10${}^{-1}$(1.83 × 10${}^{-2}$)
	5	9.6435 × 10${}^{-1}$(5.90 × 10${}^{-4}$)+	9.5697 × 10${}^{-1}$(6.41 × 10${}^{-4}$)-	9.5681 × 10${}^{-1}$(1.85 × 10${}^{-2}$)-	**9.6535 × 10${}^{-1}$(2.08 × 10${}^{-4}$)+**	9.6111 × 10${}^{-1}$(1.78 × 10${}^{-3}$)
	10	9.9912 × 10${}^{-1}$(1.35 × 10${}^{-3}$)+	9.9904 × 10${}^{-1}$(4.95 × 10${}^{-5}$)+	9.9549 × 10${}^{-1}$(6.71 × 10${}^{-3}$)-	**9.9947 × 10${}^{-1}$(1.94 × 10${}^{-5}$)+**	9.9891 × 10${}^{-1}$(1.22 × 10${}^{-3}$)
	15	9.9929 × 10${}^{-1}$(6.53 × 10${}^{-4}$)-	**9.9997 × 10${}^{-1}$(5.39 × 10${}^{-6}$)+**	9.9924 × 10${}^{-1}$(1.62 × 10${}^{-3}$)-	9.9975 × 10${}^{-1}$(7.88 × 10${}^{-4}$)+	9.9942 × 10${}^{-1}$(2.61 × 10${}^{-3}$)
+/−/=		4/11/1	3/11/2	2/12/2	6/8/2	

^1^ “+”, “−”, or “=” indicates that the corresponding algorithm is significantly better than, worse than, or comparable to DP-NSGA-III. The best average value among these algorithms on each test problem is highlighted in bold.

**Table A3:** IGD values obtained by DP-NSGA-III and other peer algorithms in the DC-DTLZ suite ^1^.

Problem	*M*	NSGA-III	C-TAEA	DCNSGA-III	MOEA/D-2WA	DP-NSGA-III
DC1DTLZ1	3	1.4149 × 10${}^{-2}$(1.95 × 10${}^{-4}$)-	1.5618 × 10${}^{-2}$(4.13 × 10${}^{-4}$)-	1.3807 × 10${}^{-2}$(1.95 × 10${}^{-4}$)-	2.7167 × 10${}^{-2}$(5.37 × 10${}^{-3}$)-	**1.3601 × 10${}^{-2}$(1.51 × 10${}^{-4}$)**
	5	**3.9846 × 10${}^{-2}$(1.65 × 10${}^{-4}$)+**	4.2228 × 10${}^{-2}$(4.69 × 10${}^{-4}$)-	4.0444 × 10${}^{-2}$(2.61 × 10${}^{-4}$)-	4.3698 × 10${}^{-2}$(3.61 × 10${}^{-5}$)-	4.0141 × 10${}^{-2}$(1.91 × 10${}^{-4}$)
	10	8.6732 × 10${}^{-2}$(3.27 × 10${}^{-3}$)-	9.6071 × 10${}^{-2}$(2.55 × 10${}^{-3}$)-	8.8756 × 10${}^{-2}$(1.35 × 10${}^{-2}$)=	**7.9532 × 10${}^{-2}$(1.44 × 10${}^{-4}$)+**	8.5734 × 10${}^{-2}$(1.12 × 10${}^{-3}$)
	15	1.2823 × 10${}^{-1}$(1.99 × 10${}^{-5}$)=	1.4122 × 10${}^{-1}$(5.93 × 10${}^{-3}$)-	1.3728 × 10${}^{-1}$(9.13 × 10${}^{-3}$)-	**1.2557 × 10${}^{-1}$(7.74 × 10${}^{-4}$)+**	1.2736 × 10${}^{-1}$(1.97 × 10${}^{-3}$)
DC1DTLZ3	3	4.6098 × 10${}^{-2}$(9.37 × 10${}^{-4}$)-	4.4586 × 10${}^{-2}$(1.17 × 10${}^{-3}$)=	4.7993 × 10${}^{-2}$(1.13 × 10${}^{-2}$)-	4.6526 × 10${}^{-2}$(7.32 × 10${}^{-3}$)-	**4.4474 × 10${}^{-2}$(1.04 × 10${}^{-3}$)**
	5	1.4445 × 10${}^{-1}$(6.38 × 10${}^{-4}$)-	2.2595 × 10${}^{-1}$(2.12 × 10${}^{-2}$)-	1.4455 × 10${}^{-1}$(8.29 × 10${}^{-4}$)-	1.4340 × 10${}^{0}$(7.70 × 10${}^{-1}$)-	**1.4325 × 10${}^{-1}$(9.35 × 10${}^{-4}$)**
	10	5.0342 × 10${}^{-1}$(4.18 × 10${}^{-2}$)-	5.1141 × 10${}^{-1}$(2.40 × 10${}^{-2}$)-	4.8251 × 10${}^{-1}$(4.40 × 10${}^{-2}$)-	**3.9529 × 10${}^{-1}$(4.18 × 10${}^{-3}$)+**	4.0617 × 10${}^{-1}$(6.62 × 10${}^{-3}$)
	15	7.2158 × 10${}^{-1}$(5.90 × 10${}^{-2}$)-	7.1515 × 10${}^{-1}$(3.52 × 10${}^{-2}$)-	7.0274 × 10${}^{-1}$(4.72 × 10${}^{-2}$)-	6.1623 × 10${}^{-1}$(6.01 × 10${}^{-2}$)=	**6.0734 × 10${}^{-1}$(1.82 × 10${}^{-2}$)**
DC2DTLZ1	3	1.4465 × 10${}^{-1}$(6.13 × 10${}^{-2}$)-	2.3327 × 10${}^{-2}$(1.95 × 10${}^{-4}$)-	6.9379 × 10${}^{-2}$(7.56 × 10${}^{-2}$)-	2.0561 × 10${}^{-2}$(8.29 × 10${}^{-6}$)=	**2.0559 × 10${}^{-2}$(6.25 × 10${}^{-6}$)**
	5	1.4000 × 10${}^{-1}$(3.94 × 10${}^{-2}$)-	6.1657 × 10${}^{-2}$(3.61 × 10${}^{-4}$)-	8.0926 × 10${}^{-2}$(5.34 × 10${}^{-2}$)-	5.2707 × 10${}^{-2}$(3.66 × 10${}^{-6}$)-	**5.2706 × 10${}^{-2}$(4.76 × 10${}^{-6}$)**
	10	1.7004 × 10${}^{-1}$(2.17 × 10${}^{-2}$)-	1.4388 × 10${}^{-1}$(8.15 × 10${}^{-4}$)-	1.1874 × 10${}^{-1}$(2.31 × 10${}^{-2}$)-	1.1113 × 10${}^{-1}$(1.19 × 10${}^{-2}$)-	**1.0951 × 10${}^{-1}$(3.82 × 10${}^{-5}$)**
	15	2.7788 × 10${}^{-1}$(3.77 × 10${}^{-2}$)-	2.1802 × 10${}^{-1}$(4.29 × 10${}^{-3}$)-	1.9029 × 10${}^{-1}$(2.61 × 10${}^{-2}$)-	2.3111 × 10${}^{-1}$(5.32 × 10${}^{-2}$)=	**1.8368 × 10${}^{-1}$(6.14 × 10${}^{-4}$)**
DC2DTLZ3	3	5.6067 × 10${}^{-1}$(2.37 × 10${}^{-4}$)-	6.3740 × 10${}^{-2}$(1.24 × 10${}^{-3}$)-	5.3745 × 10${}^{-1}$(1.56 × 10${}^{-1}$)-	3.7557 × 10${}^{-1}$(2.48 × 10${}^{-1}$)-	**5.4472 × 10${}^{-2}$(8.30 × 10${}^{-6}$)**
	5	5.9661 × 10${}^{-1}$(4.30 × 10${}^{-4}$)-	1.8737 × 10${}^{-1}$(1.98 × 10${}^{-3}$)-	3.4857 × 10${}^{-1}$(2.31 × 10${}^{-1}$)-	1.6513 × 10${}^{-1}$(1.04 × 10${}^{-5}$)=	**1.6513 × 10${}^{-1}$(1.11 × 10${}^{-5}$)**
	10	8.3041 × 10${}^{-1}$(3.53 × 10${}^{-2}$)-	4.3599 × 10${}^{-1}$(3.26 × 10${}^{-3}$)-	5.9260 × 10${}^{-1}$(1.94 × 10${}^{-1}$)-	**4.2079 × 10${}^{-1}$(4.12 × 10${}^{-4}$)+**	4.2125 × 10${}^{-1}$(1.40 × 10${}^{-4}$)
	15	1.0035 × 10${}^{0}$(1.64 × 10${}^{-2}$)-	6.4346 × 10${}^{-1}$(2.09 × 10${}^{-2}$)-	8.6106 × 10${}^{-1}$(1.69 × 10${}^{-1}$)-	6.6804 × 10${}^{-1}$(1.19 × 10${}^{-1}$)-	**6.2027 × 10${}^{-1}$(4.59 × 10${}^{-4}$)**
DC3DTLZ1	3	2.2305 × 10${}^{-1}$(1.61 × 10${}^{-1}$)-	**9.9253 × 10${}^{-3}$(5.08 × 10${}^{-4}$)=**	1.0108 × 10${}^{-2}$(3.19 × 10${}^{-4}$)=	2.0631 × 10${}^{-2}$(6.73 × 10${}^{-3}$)-	9.9561 × 10${}^{-3}$(3.22 × 10${}^{-4}$)
	5	8.8082 × 10${}^{-2}$(5.76 × 10${}^{-2}$)-	3.3210 × 10${}^{-2}$(6.29 × 10${}^{-3}$)-	2.5873 × 10${}^{-2}$(1.13 × 10${}^{-2}$)=	5.9310 × 10${}^{-2}$(1.62 × 10${}^{-2}$)-	**2.2646 × 10${}^{-2}$(8.55 × 10${}^{-4}$)**
DC3DTLZ3	3	1.5363 × 10${}^{0}$(4.87 × 10${}^{-1}$)-	4.2066 × 10${}^{-2}$(1.63 × 10${}^{-2}$)=	6.2999 × 10${}^{-1}$(2.56 × 10${}^{-1}$)-	5.8570 × 10${}^{-1}$(1.32 × 10${}^{-1}$)-	**3.0312 × 10${}^{-2}$(1.72 × 10${}^{-3}$)**
	5	9.5209 × 10${}^{-1}$(4.83 × 10${}^{-1}$)-	1.0169 × 10${}^{-1}$(1.03 × 10${}^{-2}$)-	3.1576 × 10${}^{-1}$(2.48 × 10${}^{-1}$)-	2.4876 × 10${}^{-1}$(1.25 × 10${}^{-1}$)-	**8.7801 × 10${}^{-2}$(2.90 × 10${}^{-3}$)**
	10	1.6601 × 10${}^{0}$(6.41 × 10${}^{-1}$)-	1.8491 × 10${}^{-1}$(6.92 × 10${}^{-2}$)-	6.3620 × 10${}^{-1}$(3.84 × 10${}^{-1}$)-	4.9978 × 10${}^{-1}$(6.75 × 10${}^{-2}$)-	**1.2029 × 10${}^{-1}$(9.22 × 10${}^{-3}$)**
	15	3.0696 × 10${}^{0}$(1.00 × 10${}^{0}$)-	6.2199 × 10${}^{-1}$(3.64 × 10${}^{-1}$)-	7.5702 × 10${}^{-1}$(4.11 × 10${}^{-1}$)-	7.3173 × 10${}^{-1}$(2.68 × 10${}^{-1}$)-	**1.4118 × 10${}^{-1}$(9.31 × 10${}^{-3}$)**
+/−/=		1/20/1	0/19/3	0/19/3	4/14/4	

^1^ “+”, “−”, or “=” indicates that the corresponding algorithm is significantly better than, worse than, or comparable to DP-NSGA-III. The best average value among these algorithms on each test problem is highlighted in bold.

**Table A4:** HV values obtained by DP-NSGA-III and other peer algorithms in the DC-DTLZ suite ^1^.

Problem	*M*	NSGA-III	C-TAEA	DCNSGA-III	MOEA/D-2WA	DP-NSGA-III
DC1DTLZ1	3	**6.2908 × 10${}^{-1}$(1.80 × 10${}^{-3}$)+**	6.2624 × 10${}^{-1}$(1.18 × 10${}^{-3}$)+	6.2456 × 10${}^{-1}$(2.30 × 10${}^{-3}$)=	5.9725 × 10${}^{-1}$(1.81 × 10${}^{-2}$)-	6.2544 × 10${}^{-1}$(1.51 × 10${}^{-3}$)
	5	**7.7284 × 10${}^{-1}$(8.57 × 10${}^{-4}$)+**	7.6956 × 10${}^{-1}$(8.87 × 10${}^{-4}$)-	7.6977 × 10${}^{-1}$(7.37 × 10${}^{-4}$)-	7.6525 × 10${}^{-1}$(1.74 × 10${}^{-4}$)-	7.7057 × 10${}^{-1}$(1.18 × 10${}^{-3}$)
	10	7.9618 × 10${}^{-1}$(2.24 × 10${}^{-4}$)-	7.9609 × 10${}^{-1}$(1.14 × 10${}^{-4}$)-	7.9345 × 10${}^{-1}$(1.66 × 10${}^{-2}$)-	7.5413 × 10${}^{-1}$(5.97 × 10${}^{-3}$)-	**7.9681 × 10${}^{-1}$(1.11 × 10${}^{-4}$)**
	15	7.9782 × 10${}^{-1}$(7.60 × 10${}^{-6}$)-	7.9700 × 10${}^{-1}$(5.69 × 10${}^{-4}$)-	7.9658 × 10${}^{-1}$(2.55 × 10${}^{-3}$)-	6.3463 × 10${}^{-1}$(2.12 × 10${}^{-2}$)-	**7.9784 × 10${}^{-1}$(2.01 × 10${}^{-5}$)**
DC1DTLZ3	3	4.6783 × 10${}^{-1}$(1.08 × 10${}^{-3}$)=	4.5876 × 10${}^{-1}$(2.86 × 10${}^{-3}$)-	4.6354 × 10${}^{-1}$(2.02 × 10${}^{-2}$)=	**4.6891 × 10${}^{-1}$(9.39 × 10${}^{-3}$)+**	4.6701 × 10${}^{-1}$(1.88 × 10${}^{-3}$)
	5	7.6344 × 10${}^{-1}$(1.04 × 10${}^{-3}$)=	6.8741 × 10${}^{-1}$(2.11 × 10${}^{-2}$)-	7.6321 × 10${}^{-1}$(8.69 × 10${}^{-4}$)-	5.6919 × 10${}^{-3}$(1.88 × 10${}^{-2}$)-	**7.6382 × 10${}^{-1}$(9.38 × 10${}^{-4}$)**
	10	8.6557 × 10${}^{-1}$(9.56 × 10${}^{-2}$)-	9.0533 × 10${}^{-1}$(1.34 × 10${}^{-2}$)-	8.8507 × 10${}^{-1}$(6.10 × 10${}^{-2}$)-	**9.6234 × 10${}^{-1}$(6.81 × 10${}^{-4}$)+**	9.6086 × 10${}^{-1}$(1.77 × 10${}^{-3}$)
	15	7.1236 × 10${}^{-1}$(2.05 × 10${}^{-1}$)-	7.6602 × 10${}^{-1}$(2.15 × 10${}^{-1}$)-	7.5661 × 10${}^{-1}$(1.87 × 10${}^{-1}$)-	9.6225 × 10${}^{-1}$(1.39 × 10${}^{-1}$)-	**9.7225 × 10${}^{-1}$(1.22 × 10${}^{-2}$)**
DC2DTLZ1	3	5.2980 × 10${}^{-1}$(1.50 × 10${}^{-1}$)-	8.3802 × 10${}^{-1}$(3.19 × 10${}^{-4}$)-	7.1724 × 10${}^{-1}$(1.93 × 10${}^{-1}$)-	8.4167 × 10${}^{-1}$(1.11 × 10${}^{-4}$)=	**8.4168 × 10${}^{-1}$(9.28 × 10${}^{-5}$)**
	5	8.5826 × 10${}^{-1}$(5.50 × 10${}^{-2}$)-	9.7691 × 10${}^{-1}$(2.39 × 10${}^{-4}$)-	9.3819 × 10${}^{-1}$(8.13 × 10${}^{-2}$)-	9.7987 × 10${}^{-1}$(1.24 × 10${}^{-4}$)=	**9.7989 × 10${}^{-1}$(1.22 × 10${}^{-4}$)**
	10	9.7880 × 10${}^{-1}$(7.28 × 10${}^{-3}$)-	9.9944 × 10${}^{-1}$(2.90 × 10${}^{-5}$)-	9.9753 × 10${}^{-1}$(7.40 × 10${}^{-3}$)=	9.9893 × 10${}^{-1}$(4.09 × 10${}^{-3}$)=	**9.9968 × 10${}^{-1}$(1.55 × 10${}^{-5}$)**
	15	9.7478 × 10${}^{-1}$(1.01 × 10${}^{-2}$)-	9.9978 × 10${}^{-1}$(8.11 × 10${}^{-5}$)-	9.9493 × 10${}^{-1}$(1.60 × 10${}^{-2}$)=	9.8577 × 10${}^{-1}$(1.44 × 10${}^{-2}$)-	**9.9992 × 10${}^{-1}$(9.13 × 10${}^{-6}$)**
DC2DTLZ3	3	8.1376 × 10${}^{-3}$(1.24 × 10${}^{-4}$)-	5.4647 × 10${}^{-1}$(1.30 × 10${}^{-3}$)-	4.8563 × 10${}^{-2}$(1.47 × 10${}^{-1}$)-	2.1007 × 10${}^{-1}$(2.70 × 10${}^{-1}$)-	**5.5933 × 10${}^{-1}$(2.64 × 10${}^{-4}$)**
	5	7.1752 × 10${}^{-2}$(3.92 × 10${}^{-4}$)-	7.9251 × 10${}^{-1}$(2.19 × 10${}^{-3}$)-	5.1276 × 10${}^{-1}$(3.74 × 10${}^{-1}$)-	**8.1267 × 10${}^{-1}$(3.45 × 10${}^{-4}$)=**	8.1257 × 10${}^{-1}$(4.34 × 10${}^{-4}$)
	10	2.0234 × 10${}^{-1}$(4.70 × 10${}^{-2}$)-	9.6831 × 10${}^{-1}$(1.11 × 10${}^{-3}$)-	6.6125 × 10${}^{-1}$(3.78 × 10${}^{-1}$)-	9.6980 × 10${}^{-1}$(1.76 × 10${}^{-4}$)=	**9.6981 × 10${}^{-1}$(1.25 × 10${}^{-4}$)**
	15	1.8326 × 10${}^{-2}$(1.06 × 10${}^{-2}$)-	9.4186 × 10${}^{-1}$(3.48 × 10${}^{-2}$)-	3.6589 × 10${}^{-1}$(4.69 × 10${}^{-1}$)-	8.5857 × 10${}^{-1}$(3.43 × 10${}^{-1}$)=	**9.9069 × 10${}^{-1}$(1.18 × 10${}^{-4}$)**
DC3DTLZ1	3	9.6576 × 10${}^{-2}$(1.19 × 10${}^{-1}$)-	5.2180 × 10${}^{-1}$(3.16 × 10${}^{-3}$)-	**5.2776 × 10${}^{-1}$(1.74 × 10${}^{-3}$)=**	5.0561 × 10${}^{-1}$(3.22 × 10${}^{-2}$)=	5.2768 × 10${}^{-1}$(1.33 × 10${}^{-3}$)
	5	5.4568 × 10${}^{-2}$(6.35 × 10${}^{-2}$)-	8.8297 × 10${}^{-2}$(1.19 × 10${}^{-2}$)-	1.2713 × 10${}^{-1}$(5.84 × 10${}^{-3}$)-	9.3507 × 10${}^{-2}$(1.42 × 10${}^{-2}$)-	**1.2965 × 10${}^{-1}$(1.61 × 10${}^{-3}$)**
DC3DTLZ3	3	0.0000 × 10${}^{0}$(0.00 × 10${}^{0}$)-	3.3638 × 10${}^{-1}$(2.15 × 10${}^{-2}$)-	1.2002 × 10${}^{-2}$(6.57 × 10${}^{-2}$)-	1.2125 × 10${}^{-2}$(6.64 × 10${}^{-2}$)-	**3.5909 × 10${}^{-1}$(1.89 × 10${}^{-3}$)**
	5	0.0000 × 10${}^{0}$(0.00 × 10${}^{0}$)-	5.2458 × 10${}^{-1}$(2.00 × 10${}^{-2}$)-	3.0684 × 10${}^{-1}$(2.92 × 10${}^{-1}$)-	5.2956 × 10${}^{-1}$(5.67 × 10${}^{-2}$)=	**5.7579 × 10${}^{-1}$(3.94 × 10${}^{-3}$)**
	10	0.0000 × 10${}^{0}$(0.00 × 10${}^{0}$)-	6.6668 × 10${}^{-1}$(5.64 × 10${}^{-2}$)-	2.5906 × 10${}^{-1}$(3.73 × 10${}^{-1}$)-	6.3365 × 10${}^{-1}$(4.59 × 10${}^{-2}$)-	**7.8243 × 10${}^{-1}$(7.14 × 10${}^{-3}$)**
	15	0.0000 × 10${}^{0}$(0.00 × 10${}^{0}$)-	7.4586 × 10${}^{-3}$(2.03 × 10${}^{-2}$)-	1.0836 × 10${}^{-2}$(2.96 × 10${}^{-2}$)-	8.4142 × 10${}^{-2}$(4.73 × 10${}^{-2}$)-	**1.1732 × 10${}^{-1}$(1.54 × 10${}^{-2}$)**
+/−/=		2/18/2	1/21/0	0/17/5	2/12/8	

^1^ “+”, “−”, or “=” indicates that the corresponding algorithm is significantly better than, worse than, or comparable to DP-NSGA-III. The best average value among these algorithms on each test problem is highlighted in bold.

**Table A5:** IGD values obtained by DP-NSGA-III and other peer algorithms in the MW suite. ^1^

Problem	*M*	NSGA-III	C-TAEA	DCNSGA-III	MOEA/D-2WA	DP-NSGA-III
MW4	3	**4.1535 × 10${}^{-2}$(1.08 × 10${}^{-4}$)+**	4.6663 × 10${}^{-2}$(3.46 × 10${}^{-4}$)-	4.2120 × 10${}^{-2}$(5.01 × 10${}^{-4}$)=	4.1993 × 10${}^{-2}$(9.15 × 10${}^{-4}$)+	4.2103 × 10${}^{-2}$(3.36 × 10${}^{-4}$)
	5	1.0474 × 10${}^{-1}$(2.36 × 10${}^{-4}$)+	1.2218 × 10${}^{-1}$(1.18 × 10${}^{-3}$)-	1.0500 × 10${}^{-1}$(1.69 × 10${}^{-4}$)+	**1.0430 × 10${}^{-1}$(3.57 × 10${}^{-4}$)+**	1.0521 × 10${}^{-1}$(1.78 × 10${}^{-4}$)
	10	2.1816 × 10${}^{-1}$(1.12 × 10${}^{-2}$)+	2.7641 × 10${}^{-1}$(6.24 × 10${}^{-3}$)-	2.2047 × 10${}^{-1}$(7.34 × 10${}^{-3}$)-	**2.0360 × 10${}^{-1}$(8.93 × 10${}^{-4}$)+**	2.1994 × 10${}^{-1}$(1.54 × 10${}^{-3}$)
	15	3.7194 × 10${}^{-1}$(6.44 × 10${}^{-3}$)=	4.3583 × 10${}^{-1}$(9.90 × 10${}^{-3}$)-	3.7041 × 10${}^{-1}$(4.42 × 10${}^{-3}$)=	**2.4986 × 10${}^{-1}$(1.21 × 10${}^{-3}$)+**	3.7182 × 10${}^{-1}$(6.68 × 10${}^{-3}$)
MW8	3	6.2302 × 10${}^{-2}$(3.83 × 10${}^{-2}$)-	5.5022 × 10${}^{-2}$(3.58 × 10${}^{-3}$)-	4.9781 × 10${}^{-2}$(2.19 × 10${}^{-3}$)-	5.1319 × 10${}^{-2}$(3.30 × 10${}^{-3}$)-	**4.8680 × 10${}^{-2}$(2.65 × 10${}^{-3}$)**
	5	1.5959 × 10${}^{-1}$(3.49 × 10${}^{-2}$)-	1.7035 × 10${}^{-1}$(1.87 × 10${}^{-3}$)-	1.5266 × 10${}^{-1}$(2.67 × 10${}^{-4}$)-	1.5301 × 10${}^{-1}$(4.01 × 10${}^{-4}$)-	**1.5216 × 10${}^{-1}$(3.66 × 10${}^{-4}$)**
	10	4.2660 × 10${}^{-1}$(5.27 × 10${}^{-2}$)-	4.1017 × 10${}^{-1}$(3.11 × 10${}^{-3}$)-	4.0947 × 10${}^{-1}$(4.64 × 10${}^{-2}$)-	**3.9667 × 10${}^{-1}$(2.06 × 10${}^{-3}$)+**	3.9871 × 10${}^{-1}$(1.65 × 10${}^{-3}$)
	15	6.4708 × 10${}^{-1}$(1.38 × 10${}^{-2}$)-	6.4468 × 10${}^{-1}$(2.82 × 10${}^{-3}$)-	6.4353 × 10${}^{-1}$(1.22 × 10${}^{-2}$)-	**6.0777 × 10${}^{-1}$(4.53 × 10${}^{-3}$)+**	6.2793 × 10${}^{-1}$(2.55 × 10${}^{-3}$)
MW14	3	1.3425 × 10${}^{-1}$(1.02 × 10${}^{-2}$)-	**1.1616 × 10${}^{-1}$(3.84 × 10${}^{-3}$)+**	1.3657 × 10${}^{-1}$(2.50 × 10${}^{-2}$)-	2.0790 × 10${}^{-1}$(5.07 × 10${}^{-3}$)-	1.2706 × 10${}^{-1}$(3.38 × 10${}^{-3}$)
	5	3.6249 × 10${}^{-1}$(2.95 × 10${}^{-2}$)-	**3.0987 × 10${}^{-1}$(6.06 × 10${}^{-3}$)+**	3.8139 × 10${}^{-1}$(4.39 × 10${}^{-2}$)-	4.6584 × 10${}^{-1}$(7.65 × 10${}^{-3}$)-	3.3364 × 10${}^{-1}$(2.80 × 10${}^{-2}$)
	10	1.1445 × 10${}^{0}$(2.49 × 10${}^{-2}$)-	1.4023 × 10${}^{0}$(5.04 × 10${}^{-2}$)-	1.1844 × 10${}^{0}$(2.65 × 10${}^{-2}$)-	1.4500 × 10${}^{0}$(2.06 × 10${}^{-1}$)-	**1.0925 × 10${}^{0}$(2.83 × 10${}^{-2}$)**
	15	3.2374 × 10${}^{0}$(7.09 × 10${}^{-2}$)-	3.0938 × 10${}^{0}$(1.41 × 10${}^{-1}$)-	3.3312 × 10${}^{0}$(1.33 × 10${}^{-1}$)-	3.1616 × 10${}^{0}$(7.02 × 10${}^{-2}$)-	**2.7544 × 10${}^{0}$(1.58 × 10${}^{-1}$)**
+/−/=		3/8/1	2/10/0	1/9/2	6/6/0	

^1^ “+”, “−”, or “=” indicates that the corresponding algorithm is significantly better than, worse than, or comparable to DP-NSGA-III. The best average value among these algorithms on each test problem is highlighted in bold.

**Table A6:** HV values obtained by DP-NSGA-III and other peer algorithms in the C-DTLZ suite ^1^.

Problem	*M*	NSGA-III	C-TAEA	DCNSGA-III	MOEA/D-2WA	DP-NSGA-III
MW4	3	**8.4121 × 10${}^{-1}$(1.51 × 10${}^{-4}$)+**	8.3793 × 10${}^{-1}$(3.18 × 10${}^{-4}$)-	8.4069 × 10${}^{-1}$(4.56 × 10${}^{-4}$)=	8.4026 × 10${}^{-1}$(2.58 × 10${}^{-3}$)=	8.4076 × 10${}^{-1}$(2.82 × 10${}^{-4}$)
	5	**9.7976 × 10${}^{-1}$(1.64 × 10${}^{-4}$)+**	9.7721 × 10${}^{-1}$(2.88 × 10${}^{-4}$)-	9.7969 × 10${}^{-1}$(1.07 × 10${}^{-4}$)+	9.7957 × 10${}^{-1}$(1.51 × 10${}^{-4}$)=	9.7954 × 10${}^{-1}$(1.78 × 10${}^{-4}$)
	10	9.9955 × 10${}^{-1}$(6.84 × 10${}^{-4}$)=	9.9952 × 10${}^{-1}$(5.81 × 10${}^{-5}$)-	9.9966 × 10${}^{-1}$(7.69 × 10${}^{-5}$)=	9.6297 × 10${}^{-1}$(9.11 × 10${}^{-3}$)-	**9.9967 × 10${}^{-1}$(2.65 × 10${}^{-5}$)**
	15	9.9990 × 10${}^{-1}$(9.83 × 10${}^{-6}$)=	9.9984 × 10${}^{-1}$(2.52 × 10${}^{-5}$)-	9.9990 × 10${}^{-1}$(9.07 × 10${}^{-6}$)=	8.2556 × 10${}^{-1}$(2.54 × 10${}^{-2}$)-	**9.9991 × 10${}^{-1}$(1.00 × 10${}^{-5}$)**
MW8	3	5.0730 × 10${}^{-1}$(4.92 × 10${}^{-2}$)-	5.1953 × 10${}^{-1}$(1.50 × 10${}^{-2}$)-	5.2886 × 10${}^{-1}$(9.19 × 10${}^{-3}$)-	5.3090 × 10${}^{-1}$(1.40 × 10${}^{-2}$)=	**5.3278 × 10${}^{-1}$(1.18 × 10${}^{-2}$)**
	5	8.0433 × 10${}^{-1}$(2.12 × 10${}^{-2}$)=	7.8742 × 10${}^{-1}$(5.44 × 10${}^{-3}$)-	**8.1027 × 10${}^{-1}$(2.94 × 10${}^{-3}$)+**	8.0856 × 10${}^{-1}$(4.10 × 10${}^{-3}$)=	8.1020 × 10${}^{-1}$(2.88 × 10${}^{-3}$)
	10	9.5443 × 10${}^{-1}$(2.29 × 10${}^{-2}$)=	9.6348 × 10${}^{-1}$(2.09 × 10${}^{-3}$)-	9.6277 × 10${}^{-1}$(1.94 × 10${}^{-2}$)-	**9.6889 × 10${}^{-1}$(1.33 × 10${}^{-3}$)+**	9.6775 × 10${}^{-1}$(6.76 × 10${}^{-4}$)
	15	9.7484 × 10${}^{-1}$(1.10 × 10${}^{-2}$)-	9.4017 × 10${}^{-1}$(4.48 × 10${}^{-3}$)-	9.7489 × 10${}^{-1}$(9.28 × 10${}^{-3}$)-	9.8542 × 10${}^{-1}$(1.19 × 10${}^{-3}$)-	**9.9014 × 10${}^{-1}$(2.21 × 10${}^{-4}$)**
MW14	3	4.5894 × 10${}^{-1}$(6.58 × 10${}^{-3}$)-	4.6358 × 10${}^{-1}$(2.95 × 10${}^{-3}$)-	4.5976 × 10${}^{-1}$(1.11 × 10${}^{-2}$)-	4.3775 × 10${}^{-1}$(3.53 × 10${}^{-3}$)-	**4.6539 × 10${}^{-1}$(2.91 × 10${}^{-3}$)**
	5	3.4702 × 10${}^{-1}$(1.53 × 10${}^{-2}$)-	**3.9414 × 10${}^{-1}$(3.36 × 10${}^{-3}$)+**	3.6253 × 10${}^{-1}$(1.99 × 10${}^{-2}$)-	3.2687 × 10${}^{-1}$(2.63 × 10${}^{-3}$)-	3.8548 × 10${}^{-1}$(7.65 × 10${}^{-3}$)
	10	2.0239 × 10${}^{-1}$(4.52 × 10${}^{-3}$)-	**2.2912 × 10${}^{-1}$(4.66 × 10${}^{-3}$)+**	2.0483 × 10${}^{-1}$(3.25 × 10${}^{-3}$)-	1.5608 × 10${}^{-1}$(2.01 × 10${}^{-2}$)-	2.0902 × 10${}^{-1}$(5.06 × 10${}^{-3}$)
	15	1.5134 × 10${}^{-1}$(2.58 × 10${}^{-3}$)-	1.5365 × 10${}^{-1}$(3.57 × 10${}^{-3}$)-	1.5918 × 10${}^{-1}$(1.04 × 10${}^{-2}$)-	7.7070 × 10${}^{-2}$(1.17 × 10${}^{-2}$)-	**1.6459 × 10${}^{-1}$(3.82 × 10${}^{-3}$)**
+/−/=		2/6/4	2/10/0	2/7/3	1/7/4	

^1^ “+”, “−”, or “=” indicates that the corresponding algorithm is significantly better than, worse than, or comparable to DP-NSGA-III. The best average value among these algorithms on each test problem is highlighted in bold.
